# Additional root canal enlargement increases immediate postoperative pain: a randomized controlled trial

**DOI:** 10.1590/1807-3107bor-2025.vol39.108

**Published:** 2025-10-10

**Authors:** Aline Lima HARTER, Fábio de Almeida GOMES, Samantha Rodrigues XAVIER, Eduarda Carrera MALHÃO, Erick Miranda SOUZA, Fernanda Geraldo PAPPEN

**Affiliations:** (a) Universidade Federal de Pelotas – UFPel, Postgraduate Program in Dentistry, Dental Clinical Area, RS, Brazil; (b) Universidade de Fortaleza – Unifor, School of Dentistry, Department of Endodotics, Fortaleza, CE, Brazil.; (c) Universidade Federal do Maranhão – UFMA, Department of Dentistry II, São Luis, MA, Brazil.

**Keywords:** Pain, Postoperative, Root Canal Preparation, Clinical Trial

## Abstract

This prospective, double-blind, randomized clinical trial aimed to evaluate the effect of an additional instrument following the single-file instrumentation approach on postoperative pain. Fifty-six mandibular molars with asymptomatic apical periodontitis were randomly assigned to two groups: G1 – instrumentation using a single reciprocating file; G2 – additional enlargement. The frequency and intensity of postoperative pain were assessed at 24 h, 48 h, and 7 days after endodontic treatment using the numerical pain assessment scale (NPAS) (Mann-Whitney U test). Analgesic intake was also recorded and analyzed using chi-square or Fisher’s exact tests. The incidence of root canal filling extrusion was also evaluated (chi-square). Statistical significance was set at p < 0.05. At 24 h, the incidence of postoperative pain was higher when additional enlargement was performed (p = 0.019). The incidence of pain was similar between groups after 48 h (p = 0.121) and 7 days (p = 1.000). The intensity of pain was also higher at 24 h in Group 2 (p = 0.019), while it was similar between groups after 48 h (p = 0.177) and 7 days (p = 1.000). The frequency of analgesic intake was higher in Group 2 after 24 h (p = 0.019). The incidence of root canal filling material extrusion was similar in both groups (p = 0.181). In conclusion, additional enlargement following single-file root canal preparation resulted in a higher incidence and intensity of postoperative pain immediately after treatment (24 h), with no significant impact at 48 h and 7 days.

## Introduction

The introduction of reciprocating systems has sparked a debate on the necessity of using a series of instruments with increasing tip diameters, as traditionally recommended. The significant rise in positive clinical and scientific outcomes — such as reduced procedural errors, extended lifespan of NiTi files, time efficiency, and cost-effectiveness — compared to rotary instrument systems, has strengthened the argument for adopting single-file reciprocating systems as the standard for root canal preparation.^1,2^

This accelerated preparation represents an undeniable technical advancement; however, a clear mismatch emerges between the speed of preparation achieved by single-file mechanical systems and effective management of infection. A potentially negative consequence of rapid canal shaping achieved by single-file techniques is that even potent biocides, such as sodium hypochlorite, require adequate contact time to reach their full antimicrobial potential.^3^ Because irrigation is directly linked to the shaping process in multiple-file instrumentation techniques, reducing the number of files needed for canal shaping also decreases the volume of sodium hypochlorite and the time available to achieve effective disinfection. In this context, numerous studies have suggested that the use of endodontic instruments with larger diameters could enhance the reduction of bacterial contamination within the root canal system.^4^ This is achieved by maximizing the volume of irrigation solutions and increasing the mechanically debrided areas within the root canals,^5^ thereby ensuring effective removal of residual tissue and contaminated dentin, which ultimately influences treatment outcomes.^6^ Nevertheless, while additional enlargement following the single-file technique may enhance disinfection,^4^ studies investigating the correlation between the extent of apical extrusion and apical diameter size have yielded conflicting results.^7,8^

During root canal instrumentation, even when endodontic instruments do not extend beyond the apical foramen, dentin debris, pulp tissue fragments, microorganisms, and irrigants can extrude into the periapical tissues.^9^ This phenomenon has been directly linked to an intense acute inflammatory infiltrate, abscess formation in the periapical area,^10^ and consequently, to pain, swelling, and flare-ups.^9,11^ Based on these findings, it seems highly relevant to investigate whether increasing the root canal diameter — using an additional file after the single-file technique — will increase the risk of postoperative pain.

A PICO (problem/patient, intervention, comparison, and outcome) strategy was developed to evaluate the influence of using an additional instrument following the single-file instrumentation approach on patients’ postoperative pain. The tested null hypothesis was that the use of an incremental file would not influence the level of postoperative pain reported by patients.

## Methods

A pragmatic, prospective, single-center, parallel-group, double-blind, randomized clinical trial was designed, and the protocol was approved by the local Research Ethics Committee (CAAE: 19622819.8.0000.5317). Informed consent was obtained from all participants after a thorough explanation of the procedures and potential risks. The study protocol was registered in the www.ensaiosclinicos.gov.br (ReBEC) database (U1111-1264-7264). This study was reported following the guidelines outlined in the CONSORT statement (www.consort-statement.org).

A previous study evaluating postoperative pain after apical enlargement^8^ was used for sample size calculation (Sealed Envelope™, Exmouth House, London, UK). An alpha error of 0.05 and a beta power of 0.95 were specified, considering a median pain score of 4.78 for the group prepared two sizes larger than the initial binding file (IBF), and 7.08 for the group prepared three sizes larger than the IBF. The minimum estimated sample size to detect differences in postoperative pain between the experimental groups was calculated as n = 20 per group. Potential patient dropouts were accounted for to enhance the statistical power of the data.

Both male and female adults (≥ 18 years) were selected for endodontic treatment. All root canal treatments were performed in a single visit by three endodontic specialists, each having more than three years of experience. The operators were calibrated for patient inclusion criteria, file selection, and procedural techniques. Training was provided through a 50-minute lecture, during which the supervising professor, responsible for the research, explained the inclusion criteria and treatment protocols in detail.

The inclusion criteria consisted of mandibular molars with asymptomatic apical periodontitis, confirmed by an apical radiolucent area (periapical index ≥ 3) and absence of symptoms. The exclusion criteria were: a) teeth with extensive crown destruction that prevented rubber dam placement; b) presence of root or crown fractures; c) root resorption; d) teeth previously subjected to endodontic treatment; e) patients who had taken anti-inflammatory or analgesic medications within the past 72 h; f) patients who received antibiotic therapy within the previous 3 months; g) teeth with untreated periodontal disease, furcation lesions, or tooth mobility; and h) patients presenting systemic diseases that could interfere with bone tissue metabolism. Fifty-six patients met the inclusion criteria and were selected for this clinical trial.

Postoperative pain was investigated following two instrumentation protocols using a reciprocating system (Reciproc® VDW System, Munich, Germany) with different final instrumentation diameters. The selected teeth were randomly assigned to one of the two groups using a simple randomization procedure (www.random.org). An individual not involved in the research generated the randomization sequence. Consecutively numbered opaque envelopes containing the group allocation were used. A researcher not directly involved in the study performed the allocation. Randomization was stratified by operator, with three separate randomizations conducted in blocks of 16–17 patients each. The patients were blinded to the treatment they received.

Following the administration of an inferior alveolar nerve block (IANB) with 2% lidocaine containing 1:100,000 epinephrine (Alphacaine, DFL, RJ, Brazil), the tooth was isolated with a rubber dam and subsequently assigned to one of the instrumentation groups based on randomization.

### Group 1 – Instrumentation using a single instrument throughout root canal preparation

The Reciproc instrument was selected according to the manufacturer’s recommendations, based on an appropriate pre-operative radiograph. If the canal was partially or completely invisible on the pre-operative radiograph, it was considered narrow, and Reciproc 25 (25.08) was chosen. In cases in which the radiograph revealed a fully visible root canal from the pulp chamber to the apex, the canal was classified as either medium or large. If a #20 hand instrument could passively reach the working length (WL), the canal was considered medium, and Reciproc 40 (40.06) was selected for root canal shaping. If only a #15 or smaller hand instrument could passively reach the WL, Reciproc 25 (25.08) was chosen.

The selected instrument was advanced into the canal space and moved in a slow, gentle in-and-out pecking motion with an amplitude limit of no more than 3 mm. After three complete pecking motions, the instrument was removed from the canal, and its flutes were cleaned. Root canal irrigation was then performed. This sequence was repeated until the instrument reached the WL. Once the WL was reached, the shaping phase was considered complete for this group.

### Group 2 – Instrumentation with an additional instrument for root canal enlargement:

As in Group 1, the initial Reciproc instrument was selected according to the manufacturer’s recommendations, based on the root canal anatomy. The root canal preparation with the initial Reciproc file was performed in the same manner as in the previous group; however, a subsequent larger file was added to the preparation. For instance, in cases in which Reciproc R25 (25.08) was selected, additional preparation was performed using Reciproc R40 (40.06). In cases in which Reciproc R40 (40.06) was selected, additional enlargement was carried out using Reciproc R50 (50.05).

For both groups, if a #30 hand instrument could passively reach the WL, the canal was classified as large, and Reciproc 50 (50.05) was selected for instrumentation. Nevertheless, in the absence of reciprocating files larger than R50, these teeth were excluded from the study.

The files were activated using the Reciproc® Silver electric motor (VDW GmbH) with the RECIPROC ALL setting as predefined by the manufacturer. Root canal shaping was performed using a pecking motion. The WL was determined using an electronic apex locator (RomiApex A-15 – Romidan Dental Solutions, Israel) and maintained 1 mm short of the apex. A #15 K-file (Dentsply Maillefer, Ballaigues, Switzerland) was used to ensure apical patency.

During root canal shaping, 2.5% sodium hypochlorite (NaOCl) was delivered using disposable syringes and a 27-G NaviTip needle (Ultradent Products Inc., South Jordan, USA), inserted into the canal up to 3 mm short of the WL. Irrigation was performed after every three in-and-out motions when the file was removed for flute cleaning. The canal was then copiously irrigated with 2 mL of NaOCl in Groups 1 and 2. The irrigation protocol was designed so that each root canal received 20 mL of NaOCl solution. Subsequently, 3 mL of 17% EDTA was used for 3 min to remove the smear layer, followed by a final irrigation with 5 mL of 2.5% NaOCl in both groups.

The canals were dried with absorbent paper points and obturated with gutta-percha (Reciproc, VDW) and a resin-based sealer (AH Plus, Dentsply Sirona Tulsa Dental) using the single-cone technique. The access cavity was restored with resin composite, and occlusal adjustments were made to eliminate any interferences. No medication was prescribed, but all patients were given postoperative instructions to take analgesics (400 mg ibuprofen), one tablet every 6 h, in case of pain. In the event of severe postoperative pain, patients were instructed to contact the operator for further assistance.

Each patient received verbal and written explanations of the pain scale at the end of the treatment session. All participants were provided with a questionnaire to record postoperative pain at 24 h, 48 h, and 7 days after root canal treatment. Patients were instructed to complete the questionnaire at home at specified intervals. Subsequently, an evaluator (blinded to the technique used by the operator) contacted each patient to collect the data recorded on the numeric rating scale.

The numerical pain assessment scale (NPAS) consists of an 11-point scale, often presented as a series of closed boxes in ascending order of whole numbers from left to right, ranging from 0 to 10. The extreme points represent “no pain” (0) and “worst pain imaginable” (10). Analgesic intake during all evaluated periods was also recorded.

The IBM SPSS-24 statistical package (IBM SPSS Statistics, Armonk, USA) was used for statistical analysis, with statistical significance set at p < 0.05. Descriptive analyses were performed on data related to the individuals and teeth included in the study, frequency of postoperative pain, and use of postoperative medication. The mean pain scores for each group at each experimental time point (24 h, 48 h, and 7 days) were calculated by summing the scores of the patients based on their group and experimental period. Considering that the variables were not normally distributed according to the Shapiro-Wilk test, between-group comparisons were performed using Mann-Whitney U tests. The frequency of patients experiencing postoperative pain after root canal treatment and the frequency of analgesic intake were analyzed using either chi-square or Fisher’s exact tests. The frequency of root canal filling extrusion in both groups was also recorded and tested for incidence (chi-square test).

## Results

The CONSORT 2020 flowchart (Figure) illustrates the flow of participants through the different phases of the trial. This clinical trial began in November 2019, and the final sample size was completed in May 2021. Out of 62 patients assessed for eligibility, one was excluded because the diagnosis of asymptomatic apical periodontitis was not confirmed, and two declined to participate in the study. After group allocation, one patient was excluded owing to instrument fracture during instrumentation, and two others were excluded because the root canal treatment could not be completed in a single appointment. Baseline demographic data are shown in Table 1.

**Figure:**
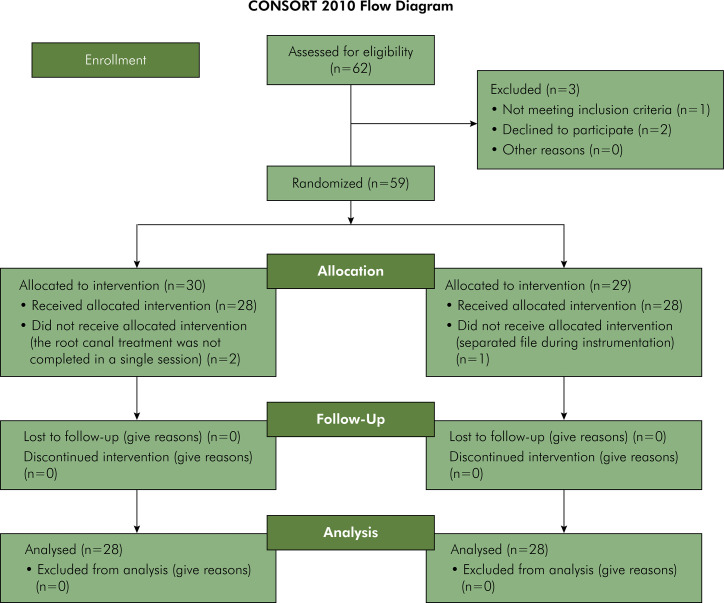
Flowchart showing sample selection, treatment, and analysis in accordance with the CONSORT statement.

**Table 1:** Baseline demographics of patients within the groups, the distribution of canal types and number of canals treated per group and incidence of root canal filling extrusion within the groups.

Variable	Group 1	Group 2	p-value
Age (mean, SD)	38.85 ± 19.42	38.17 ± 19.24	0.805^*^
Canal type n (%)			0.904^**^
First	23 (82.1)	22 (78.6)	
Second	5 (17.9)	6 (21.4)	
Number of canals treated	86	80	---
Gender n (%)			0.584^**^
Male	12 (42.9)	10 (35.7)	
Female	16 (57.1)	18 (64.3)	
Root canal filling extrusion (%)	11 (39.3)	16 (57.1)	0.181^**^

^*^One-way ANOVA; ^**^Chi-square.

This trial involved single-visit root canal treatment on 56 mandibular molars. No significant differences between the instrumentation groups were observed in terms of age (p = 0.805, chi-square) or sex (p = 0.584, chi-square). Additionally, the frequency of root canal filling material extrusion was similar in both groups (p = 0.181, chi-square).

At 24 h, the percentage of patients reporting postoperative pain was significantly higher in cases in which additional enlargement was performed (Group 2) compared with single-file instrumentation (Group 1) (p = 0.019, Fisher’s exact test). The incidence of postoperative pain was similar between the groups after 48 h (p = 0.121, Fisher’s exact test) and 7 days (p = 1.000, Fisher’s exact test) (Table 2).

**Table 2:** Incidence of postoperative pain (%) and analgesic intake among the instrumentation groups at 24h, 48h, and seven days.

Variable	Group 1	Group 2	p-value^*^
	n (%)	n (%)	
Postoperative pain			
24 hours	4 (14.3)	13 (46.4)	0.019
48 hours	4 (14.3)	10 (35.7)	0.121
7 days	0 (0.0)	1 (3.6)	1.000
Analgesic intake			
24 hours	4 (14.3)	13 (46.7)	0.019
48 hours	3 (10.7)	8 (28.6)	0.177
7 days	0 (0.0)	1 (3.6)	1.000

^*^Fisher Exact Test.

The intensity of postoperative pain in both groups is described in Table 3. The Mann-Whitney U test revealed that postoperative pain was significantly higher at 24 h in Group 2, in which additional enlargement of the root canal preparation was performed (p = 0.016). However, pain intensity was similar in both groups at 48 h (p = 0.090) and 7 days (p = 0.309).

**Table 3:** Intensity (Mean, median, IQR, Min, Max) of postoperative pain among the instrumentation groups at 24h, 48h, and seven days.

Variable	Single file instrumentation	Additional enlargement	p-value*
24 hours			
Mean (SD)	0.64 (1.49)	1.89 (2.23)	0.016
Median	0	1	
IQR	0.75	4	
Min	0	0	
Max	6	7	
48 hours			
Mean (SD)	0.43 (1.14)	1.25 (1.86)	0.090
Median	0	0	
IQR	0	3	
Min	0	0	
Max	5	5	
7 days			
Mean (SD)	0.00 (0.00)	0.04 (0.20)	0.309
Median	0	0	
IQR	-	0	
Min	0	0	
Max	0	1	

SD: standard deviation, IQR: interquartile range. * Mann-Whitney U test.

At 24 h, analgesic intake was also more frequent among patients in Group 2 (p = 0.019, Fisher’s exact test). No differences were observed between the groups after 48 h and 7 days (p = 0.177 and p = 1.000, respectively, Fisher’s exact test).

## Discussion

Postoperative pain following endodontic treatment is a clinically relevant outcome, and procedures associated with an increased risk of apical extrusion of cytotoxic organic and inorganic materials may be a primary source of postoperative pain and flare-ups.^12,13^ Therefore, controlling variables that lead to greater apical extrusion and, consequently, increased damage to periapical tissues and postoperative pain, is of paramount importance. Within the context of canal enlargement, the literature presents a dichotomy concerning its impact on factors that influence postoperative pain. Some studies report a significant reduction in debris accumulation,^14^ while others suggest that avoiding root canal enlargement prevents the extrusion of dentinal debris^15^ and filling materials.^16^ In the present study, a significant increase in the frequency and intensity of postoperative pain was observed 24 h after root canal treatment with additional enlargement compared to the single-file approach. Thus, the null hypothesis of this study was rejected, and the reasons for this outcome warrant further scrutiny.

At first glance, this outcome may be attributed to the increased extrusion of debris, as widely suggested in the literature. Pappen et al.^10^ observed that infected debris induced an intense acute inflammatory infiltrate and abscess formation, accompanied by high neutrophil infiltration, triggering an inflammatory response in the tissues. These findings align with those of other studies that describe how the pathogenic bacterial load within debris is associated with acute inflammatory reactions, mediated by increased neuropeptide expression in periapical tissues.^12^ While the hypothesis of increased debris extrusion is supported by robust inflammation-related data, postoperative pain may not be fully explained by this mechanism alone. It is more likely that the use of an additional file for diameter enlargement introduces other contributing factors that influence this outcome.

The use of an additional file led to an increased frequency of irrigation, even though the total volume of irrigant remained the same. This increased irrigation frequency may result in a longer contact time between the sodium hypochlorite (NaOCl) solution and periapical tissues, potentially leading to greater irrigation-induced inflammation and, consequently, increased postoperative pain in Group 2. Furthermore, it is well established that larger apical sizes allow for more efficient apical flow of the irrigant,^17^ promoting closer contact between the solution and periapical tissues. Therefore, the interplay between dentinal debris and irrigant extrusion should be considered when adding a file to a single-file reciprocating protocol. Unfortunately, both extruded irrigants and debris cannot be directly quantified in a clinical trial.

Root-filling extrusion is another variable that might influence postoperative pain. As a measurable variable, the frequency of root-filling extrusion was investigated in this trial. Its frequency was equally distributed between the groups, indicating no significant influence of extruded filling material, although a slightly higher number of cases was observed in Group 2. Likely, extruded filling material is not associated with an unfavorable treatment prognosis,^18^ reinforcing the observation that filling material extrusion did not influence the results of this study.

Foraminal enlargement is another instrumentation-related variable that might influence the occurrence of postoperative pain.^19^ In the present study, however, the WL was maintained 1 mm short of the apex, avoiding deliberate enlargement of the foramen, which could have resulted in a greater amount of apically extruded debris and increased contact between the irrigation solution and periapical tissues. Additionally, this trial employed a single-visit root canal treatment protocol to avoid the use of interappointment medicaments, a variable that could, by itself, influence postoperative pain immediately after the first appointment.^20^ Although previous trials have not shown differences in treatment outcomes, such as postoperative pain or success rates, when comparing single-visit and multiple-visit endodontic treatments,^21^ only cases treated with a single-visit approach were included in this study to exclude medication use as a confounding variable.

Clinical trials are generally considered a valuable source of information, as they provide real-life outcomes. Nonetheless, they inherently have limitations in controlling specific variables, as demonstrated in the present study. These limitations include: a) inability to three-dimensionally standardize the root canal anatomy of included teeth, which could result in the inclusion of hidden anatomical variations; b) lack of information on the microbial status of the specimens, which could lead to a disproportionate distribution of teeth that are more prone to microbiologically induced pain;^22^ and c) the subjective nature of pain, which is difficult to measure precisely.^11^ Items 1 and 2 refer to inclusion biases, which were controlled through randomization of a sample-size-estimated group of patients. Item 3 represents a source of methodological bias, often resulting in a limited number of studies on postoperative pain, as each patient’s pain threshold is unique and strongly influenced by cultural, individual, and economic factors.^23^

For a more reliable assessment of postoperative pain, this clinical trial employed the widely used numerical rating scale (NRS).^24^ The NRS aims for cross-cultural reliability and is considered slightly more sensitive than other tools, making it particularly useful for obtaining precise and responsive measures of pain intensity.^24^ Additionally, the numeric scale was clearly explained to each participant to ensure accurate understanding by the patient and correct interpretation by the evaluators.

According to previous studies,^25,26^ pain perception, when assessed using an NRS, can be categorized into scores: a score of 1 to 3 indicates mild pain, 4 to 6 indicates moderate pain, and 7 to 10 indicates severe pain. In the first 24 h after treatment, although the levels of pain intensity were classified as mild in both groups, a reduction of one point (or a 15.0% reduction in the NRS score) represents a minimal clinically important change (MCID) for the patient. Furthermore, a -2.0 change in the NRS score and a -33.0% change in the percent score are most closely associated with the concept of a “much better” improvement.^27^

The incidence of postoperative pain and the use of analgesics within the first 24 h after treatment were significantly higher in Group 2. Similarly, Kataia et al.^8^ demonstrated that enlargement of the apical preparation was significantly associated with higher pain scores. Contrary to the present findings, Saini et al.^19^ could not identify significant differences in the prevalence of analgesic intake or in the number of doses when comparing foraminal enlargement with conventional techniques. Ibuprofen was selected as the analgesic in this study, consistent with the recommendation from several other clinical trials, because non-steroidal anti-inflammatory drugs are the first-line treatment for postoperative pain following endodontic procedures.^23^

The reciprocating single-file approach has helped to resolve important technical issues related to the debridement efficiency and safety of NiTi-based root canal preparation, resulting in faster root canal enlargement.^1^ Whether the use of a single-file is sufficient to reach the best clinical ratio of apical healing remains an unaddressed topic. Few clinical studies have investigated the effect of enlarging the apical diameter with a focus on different outcomes, and most of them have used manual instrumentation.^19,28,29^ It is important to emphasize that only Saini et al.^19^conducted a prospective trial, while the other two studies were retrospective, in which the effect of enlargement on the success of root canal treatment was not the primary objective, resulting in various sources of bias that may mislead the interpretation of their outcomes. The results from these studies point out that enlarging the root canal may not be as advantageous for increasing the success rate as might be expected. A recently published RCT has concluded that a preparation taper of .04 and size two files larger than the initial apical binding file reduced the success rate by about 35% when compared to a taper .06 preparation with the same apical size.^30^ As the single-file Reciproc instruments used in the present trial featured tapers of .08 and .06 in the first 3 mm of the apical region, it seems unlikely that an additional file would imply a significantly higher success rate, which is certainly a subject to be further studied. Regarding postoperative pain, the primary outcome of the present study, the use of an additional instrument in the single-file Reciproc technique leads to significantly more immediate pain and analgesic intake. This serves as a compelling reason to avoid adopting this procedure unless a clear benefit in success rates is demonstrated in the future.

While this study provides valuable insights into the effects of different instrumentation protocols on postoperative pain following endodontic treatment, some limitations should be acknowledged. First, the single-center design may limit the generalizability of the findings, as patient demographics, operator expertise, and clinical practices may vary across different settings. Also, although efforts were made to standardize the procedure and calibrate the operators, variations in clinical techniques and patient anatomy could still influence the outcomes.

Reliance on patient-reported pain scores using the NPAS introduces the possibility of subjective bias, as pain perception can vary significantly among individuals. The study did not account for potential confounding factors such as psychological stress, anxiety, or previous pain experiences, all of which may influence the perception of postoperative pain. Future multicenter studies with larger sample sizes, longer follow-up periods, and broader inclusion criteria are recommended to validate these findings and enhance their generalizability. Incorporating objective measures of pain and controlling for additional confounding factors could further strengthen the robustness of future research in this area.

In conclusion, the additional enlargement of root canal preparation in mandibular molars with asymptomatic apical periodontitis resulted in higher incidence and intensity of postoperative pain and analgesic intake immediately after root canal treatment (24 h), with no impact after 48 h and 7 days.

## Data Availability

The authors declare that all data generated or analyzed during this study are included in this published article.
